# New natural compound inhibitors of PDGFRA (platelet-derived growth factor receptor α) based on computational study for high−grade glioma therapy

**DOI:** 10.3389/fnins.2022.1060012

**Published:** 2023-01-04

**Authors:** Wenzhuo Yang, Shengnan Wang, Xiangmao Zhang, Hu Sun, Menghan Zhang, Hongyu Chen, Junxiang Cui, Jinyang Li, Fei Peng, Mingqin Zhu, Bingcheng Yu, Yifan Li, Liu Yang, Wanwan Min, Mengru Xue, Lin Pan, Hao Zhu, Bo Wu, Yinghao Gu

**Affiliations:** ^1^Department of Neurosurgery, Zibo Central Hospital, Zibo, China; ^2^Department of Neurosurgery, Cancer Hospital of Sun Yat-sen University, Guangzhou, China; ^3^Department of Neurology, The First Hospital of Jilin University, Changchun, China; ^4^Department of Clinical Laboratory, The Fifth Affiliated Hospital of Xinxiang Medical College, Xinxiang, China; ^5^School of Clinical Medicine, Weifang Medical University, Weifang, China; ^6^Division of Endocrinology, Diabetes and Metabolism, Department of Medicine, Baylor College of Medicine, Houston, TX, United States; ^7^Zhongshan School of Medicine, Sun Yat-sen University, Guangzhou, China; ^8^Department of Neurosurgical Oncology, The First Hospital of Jilin University, Changchun, China; ^9^School of Clinical Medicine, Jilin University, Changchun, China; ^10^Department of Hepatology, The First Hospital of Jilin University, Changchun, China; ^11^Department of Orthopaedics, The First Hospital of Jilin University, Changchun, China

**Keywords:** high-grade glioma, PDGFRA, natural products, Imatinib, virtual screening

## Abstract

**Background:**

High-grade glioma (HGG) is a malignant brain tumor that is common and aggressive in children and adults. In the current medical paradigm, surgery and radiotherapy are the standard treatments for HGG patients. Despite this, the overall prognosis is still very bleak. Studies have shown that platelet-derived growth factor receptor α (PDGFRA) is an essential target to treat tumors and inhibiting the activity of PDGFRA can improve the prognosis of HGG. Thus, PDGFRA inhibitors are critical to developing drugs and cancer treatment.

**Objective:**

The purpose of this study was to screen lead compounds and candidate drugs with potential inhibitors against platelet-derived growth factor receptor α (PDGFRA) from the drug library (ZINC database) in order to improve the prognosis of patients with high-grade glioma (HGG).

**Materials and methods:**

In our study, we selected Imatinib as the reference drug. A series of computer-aided technologies, such as Discovery Studio 2019 and Schrodinger, were used to screen and assess potential inhibitors of PDGFRA. The first step was to calculate the LibDock scores and then analyze the pharmacological and toxicological properties. Following this, we docked the small molecules selected in the previous steps with PDGFRA to study their docking mechanism and affinity. In addition, molecular dynamics simulation was used to determine whether the ligand-PDGFRA complex was stable in nature.

**Results:**

Two novel natural compounds 1 and 2 (ZINC000008829785 and ZINC000013377891) from the ZINC database were found binding to PDGFRA with more favorable interaction energy. Also, they were predicted with less Ames mutagenicity, rodent carcinogenicity, non-developmental toxic potential, and tolerant with cytochrome P450 2D6 (CYP2D6). The dynamic simulation analysis demonstrated that ZINC000008829785-PDGFRA and ZINC000013377891-PDGFRA dimer complex had more favorable potential energy compared with Imatinib, and they can exist in natural environments stably.

**Conclusion:**

ZINC000008829785 and ZINC000013377891 might provide a solid foundation for drugs that inhibit PDGFRA in HGG. In addition to being safe drug candidates, these compounds had important implications for improving drugs targeting PDGFRA.

## 1 Introduction

High-grade glioma (HGG) is a malignant brain tumor that is common and aggressive in children and adults. In the current medical paradigm, surgery, and radiotherapy are the standard treatments for HGG patients. Temozolomide or Carmustine chips are also administered as part of the treatment regimen. Despite this, the overall prognosis is still very bleak. In general, patients survive 18 months on average, 30 percent survive 2 years, and ten percent survive 3 years or more. Because of its heterogeneity and instability, HGG is susceptible to multiple resistance to radiation and chemical treatment (Weller, 2011), and patients frequently consider targeted therapies after up-front radiation and at recurrence (Miklja et al., 2020).

Platelet-derived growth factor receptor α(PDGFRA) is one of the hot-spot targets in HGG. It is one of the most frequently altered genes in HGG. In 12% of adults with HGGs and 21% of kids with HGGs, PDGFRA is mutated or amplified. The PDGFRA receptor subunit interacts with four PDGF ligands out of two subunits in the receptor (Farahani and Xaymardan, 2015). It regulates normal glial cell proliferation and oligodendrocyte differentiation in the central nervous system (CNS) during normal development (Alentorn et al., 2012). As a result of the amplification of PDGFRA, the PI3K/mTOR signaling pathway or MAPK signaling pathway is commonly activated in HGG (Qu et al., 2010; Paugh et al., 2011; Schwartzentruber et al., 2012). Multiple cellular activities are induced, including proliferation, transformation, migration, and survival of cells (Farahani and Xaymardan, 2015). These mutations are connected to aggressive behaviors in gliomas (Koschmann et al., 2016; Mackay et al., 2018). It is therefore crucial to select PDGFRA inhibitors that are effective in cancer treatment.

Currently, the most studied PDGFRA inhibitors include Dasatinib, Avapritinib, Imatinib, and so on. Imatinib was the first tyrosine kinase inhibitor and received approval from the Food and Drug Administration (FDA) for the treatment of chronic myelogenous leukemia (Mansilla et al., 2012) and gastrointestinal stromal tumors (Gronwald et al., 1988). Imatinib is also a potent inhibitor of wild-type PDGFR family members (Wilson et al., 2018). Imatinib can induce PDGFRA phosphorylation and exert a growth inhibitory effect on glioma cells. Its efficacy against HGG had been demonstrated both *in vitro* and *in vivo* (Holtkamp et al., 2006). The drug used in the treatment of HGG has also entered the clinical trial stage. We selected Imatinib as the reference drug in this study. However, prolonged Imatinib treatment may cause mutations in PDGFRA which are Imatinib-resistant (Helbig et al., 2008). Intratumoral hemorrhage was observed in 84 recurrent pHGG patients treated with Imatinib in a phase I trial (Pollack et al., 2007). The aim of this study was to screen natural compounds from natural drugs that are more effective in treating HGG than Imatinib.

Through structural modification, natural products, such as lead compounds, can be converted into new drugs in the pharmaceutical industry (Yarla et al., 2016). To identify compounds that may have potential regulatory functions for PDGFRA from Natural Products Database, structural biological and chemical methods (including virtual screening, molecular docking, etc.) were utilized in this study. These compounds were also predicted to be absorbed, distributed, metabolized, excreted, and toxic. To develop PDGFRA inhibitors, we present a list of drug candidates and their pharmacological properties.

## 2 Materials and methods

### 2.1 Software and ZINC15 database

Discovery Studio 2019 (DS 2019) is a comprehensive modeling and simulation tool used widely in molecular biology and environmental science. Among others, it displays chemical/biological data, performs simulations/analyses, constructs three-dimensional molecules, simulates dynamic changes, and provides three-dimensional mapping. DS 2019 has been applied to a variety of life science research fields, including drug discovery, bioinformatics, structural biology, and tumor research. To screen for potential PDGFRA inhibitors, DS 2019 was applied in this study. In the first step, we screened small molecules that docked with PDGFRA using the LibDock module. The pharmacological and toxicological properties of selected compounds were also analyzed using the ADME and TOPKAT modules. We then used CDOCKER module to achieve more accurate docking between proteins and molecules. The molecular docking results were refined by using Schrodinger’s equation. In addition, small molecules were downloaded from the ZINC15 database (developed by Irwin and Shoichet Laboratories, Department of Pharmaceutical Chemistry, University of California, San Francisco, CA, USA). The ZINC15 database contains 17,931 natural, purchasable, for-sale molecules.

### 2.2 LibDock-based virtual screening

**FIGURE 1:**
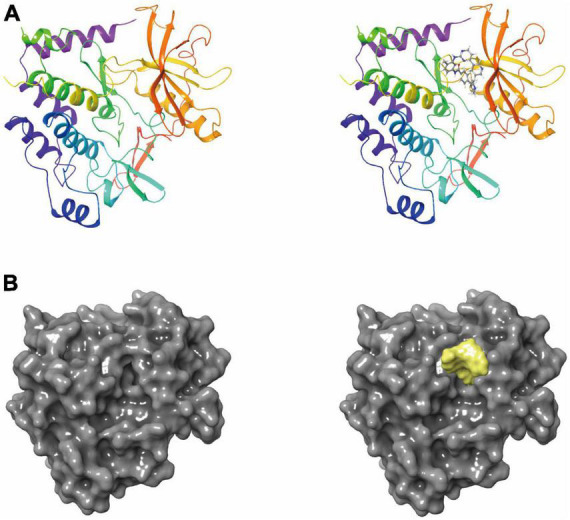
**(A)** The molecular structure of platelet-derived growth factor receptor α (PDGFRA), as well as the complex structure of PDGFRA combined with imatinib. **(B)** A molecular structure of PDGFRA and a complex structure of PDGFRA with Imatinib. Yellow represents Imatinib and gray represents PDGFRA.

Discovery Studio 2019’s LibDock module performed a rigidity-based virtual screening (Rao et al., 2007). To make proteins, hydrogen, protonation, ionization, and energy minimization are used to remove crystalline water and other heteroatoms (Chamberlain, 2010). The first step of this procedure was to calculate hotspots that characterized where the ligand interacts with PDGFRA. After the ligand formed multiple conformations, docking was performed and then the docking was optimized and scored. These conformations were docked into the receptor’s binding pocket using the principle of matching the conformation of small molecules with the receptor’s hotspot. Its main advantages were speed, parallelism, and large-scale virtual filtering. Molecule positions were ranked according to the LibDock score (Li et al., 2021a). To screen Imatinib for its ability to bind to PDGFRA, we chose the binding pocket region where it binds to PDGFRA. Crystal structures of human PDGFRA and inhibitor have been downloaded from PDB (the protein database ID: 6JOK). Figure 1 shows PDGFRA and Imatinib-PDGFRA complex’s chemical structure. Protein preparation involves removing the water of crystallization and other heteroatoms and hydrogenating, protonating, ionizing, and minimizing energy consumption. The active docking site was generated by binding the ligand Imatinib to the binding site determined by the prepared protein. LibDock then performs virtual filtering to dock molecules at the defined region. Next, all docking positions were sorted and grouped according to Lidock scores.

### 2.3 Calculation of ADME (absorption, distribution, metabolism, and excretion) and toxicity

Absorption, distribution, metabolism, and excretion module of DS 2019 was used to evaluate blood-brain barrier penetration (BBB), hepatotoxicity, CYP2D6 inhibition, plasma protein binding levels (PPB), aqueous solubility, and human intestinal absorption of molecules. We calculated molecules’ toxicological properties using DS’s TOPKAT module, including rodent carcinogenicity, developmental toxicity potential (DTP), and Ames mutagenicity (AMES) (Li et al., 2021b). When selecting potential inhibitors of PDGFRA, all of the above calculations were taken into account.

### 2.4 An analysis of CDOCKER and assessment of pharmacophores

Discovery Studio’s CDOCKER module was used for high-precision docking using the CHARMM force field. The docking conformation of LibDock’s ligand-PDGFRA is precisely re-docked by CDOCKER. Both receptors and ligands have been enhanced with CHARMM force fields. PDGFRA remains rigid in docking, whereas ligands are flexible. During the CDOCKER process, each ligand displayed ten docking postures, and the interaction energies were calculated for each pose. We selected the ligand with the highest docking score and the most appropriate direction. A CDOCKER interaction energy was calculated for each complex posture, which indicates ligand-PDGFRA affinity. Each molecule can adopt as many as 255 conformations, but only those within the energy threshold of 10 kcal/mol can survive. To further visualize the optimal binding state of the ligand and protein, Schrodinger software was used. To display compound pharmacophores, the pharmacophore formation module of 3D-QSAR was used.

### 2.5 Molecular dynamic simulation

On account of the importance of evaluating the stability of the ligand-PDGFRA complex in the natural environment, a molecular dynamics simulation module was designed. Following the above analysis, the best conformation of the ligand was further evaluated in the molecular dynamic simulation module. As a first step, we placed the ligand-receptor complex in an orthogonal box and developed a transparent periodic boundary solvated water model. Our next step is to simulate the physiological environment by adding sodium chloride with an ionic strength of 0.145. CHARMM’s force field was added to energy minimization (the steepest descent and conjugate gradient were 500 steps). For a balanced simulation of 2 ps, the system’s temperature rose slowly from 50 to 300 K. Equilibrium simulation and production module were run separately for 5 and 100 ps (Zhong et al., 2021). Production module time step was 1 fs. A particle mesh Ewald algorithm was also used to evaluate the long-range electrostatic field. In this case, the constant temperature was set at 300 K. As a result of the linear constraint solver algorithm, all hydrogen bonds were fixed. In accordance with DS’s trajectory protocol, structural characteristics, potential energy, and root-mean-square deviation’s (RMSD’s) trajectory were drawn based on the initial complex setup. The original confirmation has been obtained by molecular docking with the CDOCKER module.

## 3 Results

### 3.1 Screening inhibitors of PDGFRA virtually

**FIGURE 2:**
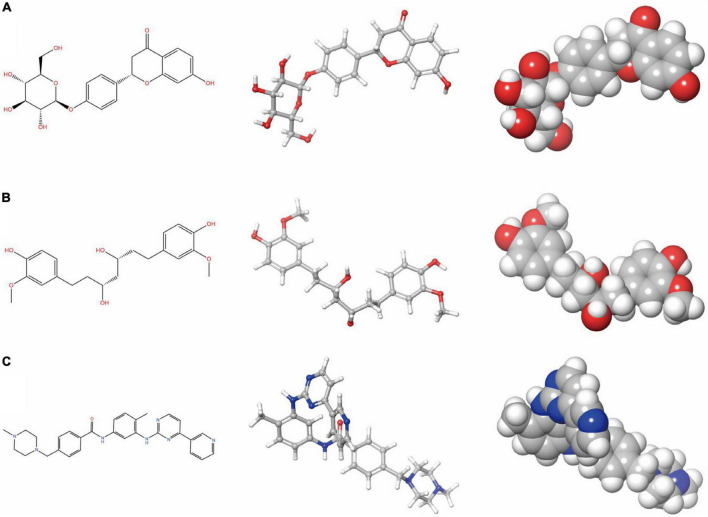
Schrodinger selected the 2D and 3D structures of Imatinib and novel compounds from virtual screening. **(A)** ZINC000008829785; **(B)** ZINC000013377891; and **(C)** Imatinib.

Platelet-derived growth factor receptor α’s ligand-binding pocket played an important role in its regulation. Therefore, this pocket area is used as the reference. PDB was used to select PDGFRA as the receptor protein. Furthermore, Imatinib was selected as the reference ligand (Figure 2C). The purpose of this study was to virtually screen PDGFRA-favorable small molecules using LibDock. There were 17,931 compounds that met the conditions of stable binding to PDGFRA, among which 3,229 compounds scored higher than Imatinib (103.14) on the LibDock test. Following are the top 20 ranked compounds (Table 1).

**TABLE 1:** Top 20 ranked compounds with LibDock scores.

Number	Compounds	LibDock score
1	ZINC000044086691	170.604
2	ZINC000004016719	157.336
3	ZINC000014780845	151.788
4	ZINC000014767731	149.908
5	ZINC000033970417	147.87
6	ZINC000004654958	145.269
7	ZINC000005762784	145.253
8	ZINC000008829785	142.413
9	ZINC000002509755	141.302
10	ZINC000004655035	141.216
11	ZINC000014658378	140.124
12	ZINC000028817821	140.061
13	ZINC000014883348	139.94
14	ZINC000013377891	139.898
15	ZINC000032840901	139.368
16	ZINC000014657833	139.198
17	ZINC000027646086	138.897
18	ZINC000003791929	138.273
19	ZINC000004557101	137.478
20	ZINC000001916008	137.366
	Imatinib	103.14

### 3.2 Prediction of pharmacological and toxicological effects

We first calculated the pharmacological properties of Imatinib and 20 ligands using the DS’s ADME module, including PPB, human intestinal absorption, BBB, hepatotoxicity, CYP2D6 inhibition, and aqueous solubility (Table 2). At 25°C, 13 compounds are predicted to be soluble in water by aqueous solubility predictions. Among them, ZINC000004654958, ZINC000008829785, ZINC000013377891, and ZINC000027646086 has improved water-solubility. CYP2D6 is an essential enzyme in drug metabolism. Except for ZINC000004016719, ZINC000014780845, ZINC000032840901, and ZINC000002509755, most compounds have no inhibitory effect on CYP2D6. In addition, in predictive hepatotoxicity, we found that 18 compounds were non-hepatotoxicity, and 2 compounds were similar in toxicity to Imatinib. 13 compounds were predicted to be higher than Imatinib in human intestinal absorption levels. Finally, 14 of the compounds were shown to have high binding to plasma proteins, while the others did not.

**TABLE 2:** Absorption, distribution, metabolism, and excretion properties of compounds.

Number	Compounds	Solubility level	BBB level	CYP2D6	Hepatotoxicity	Absorption level	PPB level
1	ZINC000044086691	1	4	0	0	3	1
2	ZINC000004016719	2	4	1	0	3	0
3	ZINC000014780845	2	4	0	1	0	1
4	ZINC000014767731	0	4	0	0	3	1
5	ZINC000033970417	1	4	0	0	3	1
6	ZINC000004654958	3	4	0	0	1	0
7	ZINC000005762784	2	1	0	0	0	1
8	ZINC000008829785	3	4	0	0	2	0
9	ZINC000002509755	2	2	1	1	0	1
10	ZINC000004655035	0	4	0	0	3	1
11	ZINC000014658378	2	0	0	0	0	1
12	ZINC000028817821	2	2	0	0	0	1
13	ZINC000014883348	0	4	0	0	3	1
14	ZINC000013377891	3	4	0	0	0	1
15	ZINC000032840901	3	4	1	0	1	0
16	ZINC000014657833	2	0	0	0	1	1
17	ZINC000027646086	4	1	0	0	0	0
18	ZINC000003791929	0	4	0	0	3	1
19	ZINC000004557101	3	4	0	0	1	0
20	ZINC000001916008	1	4	0	0	3	1
21	Imatinib	2	2	0	1	0	0

BBB, blood-brain barrier; CYP2D6, cytochrome P-450 2D6; PPB, plasma protein binding. Aqueous-solubility level: 0, extremely low; 1, very low, but possible; 2, low; 3, good. BBB level: 0, very high penetrant; 1, high; 2, medium; 3, low; 4, undefined. CYP2D6 level: 0, non-inhibitor; 1, inhibitor. Hepatotoxicity: 0, non-toxic; 1, toxic. Human-intestinal absorption level: 0, good; 1, moderate; 2, poor; 3, very poor. PPB: 0, absorbent weak; 1, absorbent strong.

To ensure the safety of these compounds, this study also conducted comprehensive research. To predict the toxicity indicators of the selected compounds and Imatinib, the TOPKAT module of DS was applied. As part of this module, three indicators were predicted, including rodent carcinogenicity, DTP, and AMES (Table 3). The results showed that 13 molecules were not mutagenic, and 9 molecules were not developmental toxic. Several studies have found that Imatinib had developmental toxicity properties and higher rodent carcinogenicity in the mouth of male rats. Two compounds were identified as potentially ideal lead compounds based on all of the above results: ZINC000008829785 (compound 1) and ZINC000013377891 (compound 2) due to lack of hepatotoxicity, CYP2D6 inhibition, AMES, rodent carcinogenicity, and developmental toxicity potential. Therefore, ZINC000008829785 and ZINC000013377891 proved safe candidates for subsequent studies (Figures 2A, B).

**TABLE 3:** Toxicities of compounds.

Number	Compounds	Mouse NTP		Rat NTP		Ames	DTP
		Female	Male	Female	Male		
1	ZINC000044086691	0.004	0.998	0.987	0	1	1
2	ZINC000004016719	0.265	0.05	1	1	0	1
3	ZINC000014780845	0	0.975	1	1	0.113	1
4	ZINC000014767731	1	0	1	1	0	1
5	ZINC000033970417	0	0.021	0	0	1	0
6	ZINC000004654958	0	0	0	0	0	0
7	ZINC000005762784	0	0.001	0	0.001	1	0
8	ZINC000008829785	0	0	0	0	0	1
9	ZINC000002509755	0.996	0.535	0	0.001	0.603	0.019
10	ZINC000004655035	1	0	1	1	0	1
11	ZINC000014658378	0	0	1	1	0	1
12	ZINC000028817821	0	0	0	0	1	0.265
13	ZINC000014883348	0	0.968	0	0	1	0
14	ZINC000013377891	0.017	0.971	0	0.008	0.122	1
15	ZINC000032840901	0.448	0.001	0	0.047	0	0
16	ZINC000014657833	1	0	1	1	0.04	1
17	ZINC000027646086	0	0	0	0	0	0
18	ZINC000003791929	1	0	1	1	0	1
19	ZINC000004557101	0	0	0	0.006	0	0
20	ZINC000001916008	1	0	1	1	0	1
21	Imatinib	0.03	0	0	1	0.102	1

NTP, U.S. national toxicology program; DTP, developmental toxicity potential. NTP <0.3 (non-carcinogen); >0.8 (carcinogen). Ames <0.3 (non-mutagen); >0.8 (mutagen). DTP <0.3 (non-toxic); >0.8 (toxic).

### 3.3 Analyses of ligand binding and ligand pharmacophores

**FIGURE 3:**
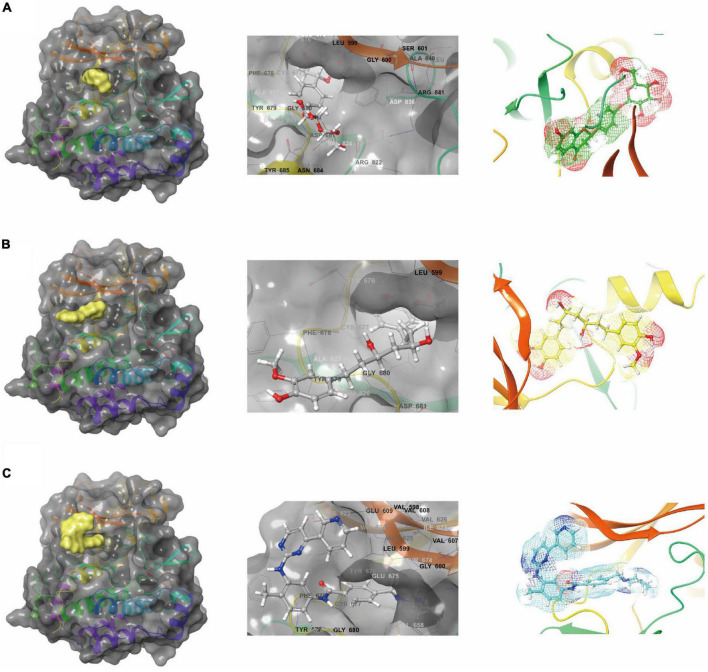
Schematic drawing of interactions between ligands and platelet-derived growth factor receptor α (PDGFRA) by Schrodinger. **(A)** ZINC000008829785-PDGFRA complex: structures and net electron cloud structures of ZINC000008829785 are shown in purple sticks. **(B)** ZINC000013377891-PDGFRA complex: structures and net electron cloud structures of ZINC000013377891 are shown in yellow sticks. **(C)** Imatinib-PDGFRA complex: structures and net electron cloud structures of Imatinib are shown in green sticks.

**FIGURE 4:**
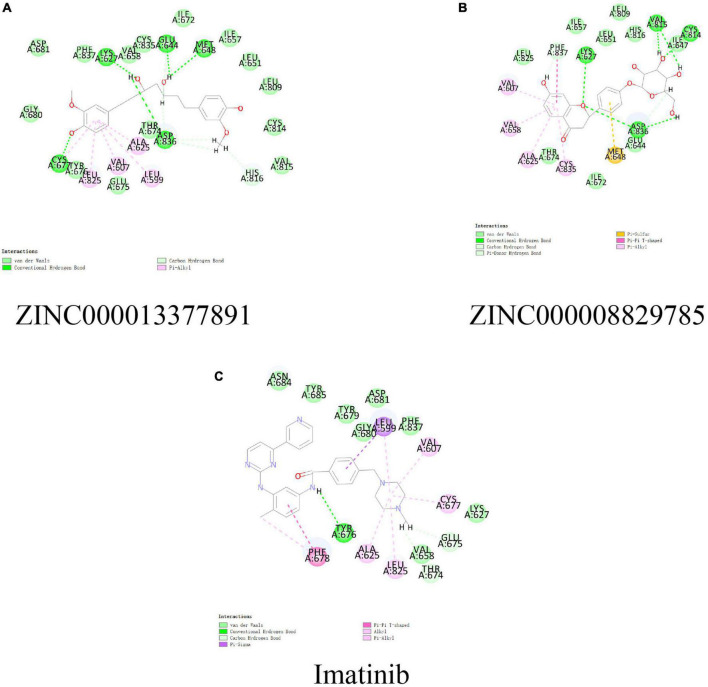
Schematic of intermolecular interaction in the binding pockets by DS of the predicted binding modes of **(B)** ZINC000008829785 with platelet-derived growth factor receptor α (PDGFRA), **(A)** ZINC000013377891 with PDGFRA, and **(C)** Imatinib with PDGFRA.

In conjunction with the CHARMm36 force field, the CDOCKER module docked the ligand precisely into the PDGFRA. We studied the interaction mechanism of Imatinib, ZINC000008829785, and ZINC000013377891 with PDGFRA, including bond type, bond length, and CDOCKER potential energy. CDOCKER potential energy is shown in Table 4. Compared with the reference ligand Imatinib (−34.6412 kcal/mol), the CDOCKER potential energy of ZINC000008829785 and ZINC000013377891 was lower, indicating that the binding ability of these two molecules to PDGFRA was superior to that of Imatinib. In addition, we applied structural calculation methods to analyze the interaction relationships formed by ligand-PDGFRA complexes (Figure 3), such as hydrogen bonds, and hydrophobic interactions (Alkyl interactions, Pi-Alkyl interactions, and Pi-Sigma interactions). The results are described below, 11 pair of hydrogen bonds was formed between ZINC000013377891 and PDGFRA, by the O9 of the compound with A: LYS627:HZ1 of 6JOK, O27 of the compound with A: CYS677:HN of 6JOK, O9 of the compound with A: LYS627:HE2 of 6JOK, et al. Also, five pairs of Pi-Alkyl interactions were presented in the complex. For ZINC000008829785, there were five pairs of Pi-Alkyl interactions and a pair of Pi-Pi T-shaped interactions with PDGFRA. There were also eight pairs of hydrogen bonds in the complex (A:LYS627:HZ–ZINC000008829785:O23, A:CYS814:HG–ZINC000008829785:O18, A:ASP836:HN–ZINC 000008829785:O23, ZINC000008829785:H40–A:ASP836:OD1, ZINC000008829785:H42–A:VAL815:O, ZINC000008829785:H 44–A:VAL815:O, ZINC000008829785:H37–A:ASP836:OD1, and ZINC000008829785:H50–A:PHE837). About the reference compound Imatinib, it formed three pairs of hydrogen bonds with PDGFRA (Molecular:H38–A:TYR676:OH, Molecular:H51–A:GLU675:O, and Molecular:H52–A:THR674 :OG1). A total of two pairs of Pi-Alkyl interaction, 1 Pi-Sigma interaction, 1 Pi-Pi T-shaped interaction, and 5 Alkyl interactions were also formed with PDGFRA (Tables 5, 6). These binding interactions were further analyzed using Schrodinger (Figure 4). The green dashed line represents hydrogen bonds, and the more hydrogen bonds, the higher the binding affinity. In conclusion, these results imply that ZINC000008829785 and ZINC000013377891 may have a better binding affinity with PDGFRA than Imatinib, indicating the promising application of these two compounds.

**TABLE 4:** CDOCKER Potential energy of compounds with platelet-derived growth factor receptor α (PDGFRA).

Compound	-CDOCKER potential energy (kcal/mol)
ZINC000008829785	44.7761
ZINC000013377891	45.2444
imatinib	34.6412

**TABLE 5:** Hydrogen bond interaction parameters for each compound with platelet-derived growth factor receptor α (PDGFRA).

Receptor	Compound	Donor atom	Receptor atom	Distances (Å)
6JOK	ZINC000013377891	A:LYS627:HZ1	ZINC000013377891:O9	2.11092
		A:CYS677:HN	ZINC000013377891:O27	1.93733
		ZINC000013377891:H37	A:ASP836:O	2.88582
		ZINC000013377891:H41	A:GLU644:OE1	2.0075
		ZINC000013377891:H41	A:MET648:SD	2.74568
		A:LYS627:HE2	ZINC000013377891:O9	2.73758
		ZINC000013377891:H40	A:GLU644:OE1	2.61332
		ZINC000013377891:H40	A:ASP836:O	2.62855
		ZINC000013377891:H49	A:HIS816:O	2.49461
		ZINC000013377891:H50	A:ASP836:OD1	2.61296
		ZINC000013377891:H51	A:ASP836:OD1	3.07523
		A:LYS627:HZ1	ZINC000008829785:O23	2.88712
	ZINC000008829785	A:CYS814:HG	ZINC000008829785:O18	2.19944
		A:ASP836:HN	ZINC000008829785:O23	2.47838
		ZINC000008829785:H40	A:ASP836:OD1	2.63525
		ZINC000008829785:H42	A:VAL815:O	2.88983
		ZINC000008829785:H44	A:VAL815:O	1.83837
		ZINC000008829785:H37	A:ASP836:OD1	2.46762
		ZINC000008829785:H50	A:PHE837	2.69054
		Imatinib:H38	A:TYR676:OH	2.81239
	Imatinib	Imatinib:H51	A:GLU675:O	2.30423
		Imatinib:H52	A:THR674:OG1	2.89619

**TABLE 6:** Hydrophobic interaction parameters for each compound with platelet-derived growth factor receptor α (PDGFRA).

Compound	Hydrophobic bond type	Donor atom	Receptor atom	Distances (Å)
	Pi-Alkyl	ZINC000013377891	A:LEU599	5.48452
	Pi-Alkyl	ZINC000013377891	A:VAL607	5.25537
ZINC000013377891	Pi-Alkyl	ZINC000013377891	A:ALA625	3.58356
	Pi-Alkyl	ZINC000013377891	A:CYS677	5.19166
	Pi-Alkyl	ZINC000013377891	A:LEU825	4.45943
	Pi-Pi T-shaped	A:PHE837	ZINC000008829785	5.50749
	Pi-Alkyl	ZINC000008829785	A:MET648	4.4137
	Pi-Alkyl	ZINC000008829785	A:VAL607	4.4684
ZINC000008829785	Pi-Alkyl	ZINC000008829785	A:ALA625	5.34806
	Pi-Alkyl	ZINC000008829785	A:VAL658	5.22524
	Pi-Alkyl	ZINC000008829785	A:CYS835	4.66191
	Pi-Sigma	A:LEU599:CD1	Molecule	3.72166
	Pi-Pi T-shaped	A:PHE678	Molecule	4.43744
	Alkyl	A:LEU599	Molecule	4.74205
	Alkyl	A:VAL607	Molecule	5.18751
Imatinib	Alkyl	A:ALA625	Molecule	4.5518
	Alkyl	A:CYS677	Molecule	4.94212
	Alkyl	A:LEU825	Molecule	4.48423
	Pi-Alkyl	A:PHE678	Molecule:C28	5.15591
	Pi-Alkyl	Imatinib	A:LYS688	3.92448

**FIGURE 5:**
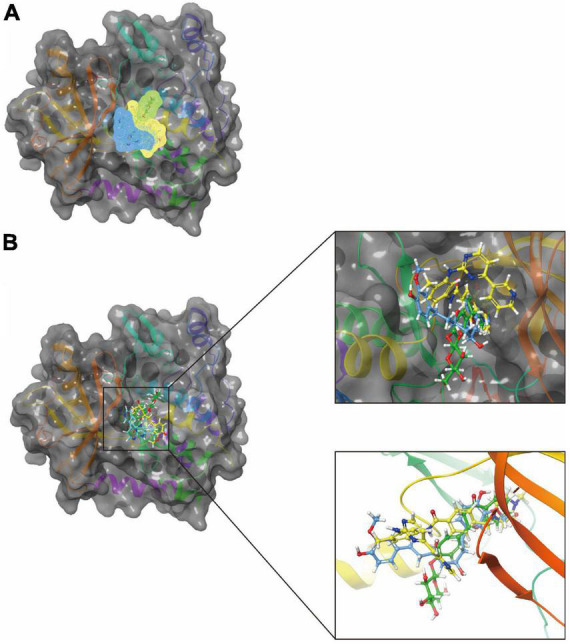
A comparison of the spatial conformation of small molecules in protein binding pockets with the gray surface of platelet-derived growth factor receptor α (PDGFRA). **(A)** In yellow, green, and red, respectively, are the structures and net electron cloud structures of ZINC000008829785, ZINC000013377891, and Imatinib. **(B)** Structures of ZINC000008829785, ZINC000013377891, and Imatinib are shown in yellow, green, and red sticks, respectively.

In addition, with the help of other virtual docking software (Schrodinger software), the conformation of ligand binding pocket in PDGFRA and the 2D and 3D structure of ligand interaction with PDGFRA amino acid residues were further demonstrated and analyzed (Figure 5). We can intuitively find that the posture of the three small molecules in the binding pocket has certain similarities. Interestingly, we found the same amino acid in the bond with PDGFRA in all three drugs. ZINC000008829785 and Imatinib form bonds with the same amino acids in protein binding pockets, including VAL-607 and ALA-625. Similarly, ZINC000013377891 and Imatinib have the same bonds in the protein binding pocket, including VAL-607, ALA-625, CYS-677, LEU-825, and LEU-599. Notably, all three molecules form the same bond with the amino acids VAL-607 and ALA-625 in the binding pocket. This phenomenon partly supports the similar inhibition of PDGFRA by the two selected small molecules and Imatinib because of their similar binding and interaction patterns. Furthermore, amino acid residues VAL-607 and ALA-625 play an important structural and functional role in the PDGFRA binding pocket domain.

**FIGURE 6:**
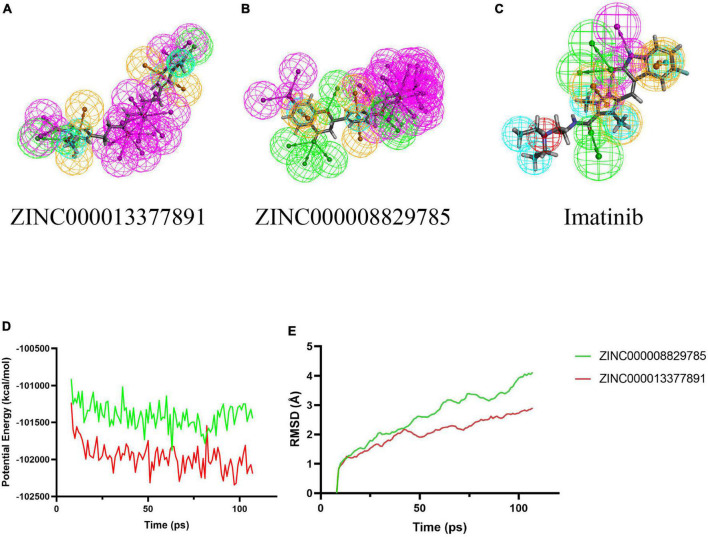
3D-QSAR module of DS used for pharmacophore prediction. By DS, green represents hydrogen acceptor; blue represents hydrophobic center; purple represents hydrogen donor; yellow represents aromatic ring. **(B)** ZINC000008829785; **(A)** ZINC000013377891; and **(C)** Imatinib. **(D,E)** Results of molecular dynamics simulation of the compounds ZINC000008829785 and ZINC000013377891. **(D)** Potential energy. **(E)** Average backbone root-mean-square deviation. RMSD, root-mean-square deviation.

As for the pharmacophore of these two compounds, the results showed 58 characteristic pharmacophores in ZINC000008829785 and 42 characteristic pharmacophores in ZINC000013377891 (Table 7). In addition, Figures 6A–C shows the hydrogen bond receptor, hydrogen bond donor, and hydrophobic center in ZINC000008829785 and ZINC000013377891.

**TABLE 7:** The analysis of feature pharmacophores.

	Total	HB_acceptor	HB_donor	Hydrophobic	Ring_aromatic	Pos_ionizable
ZINC000013377891	42	18	16	4	4	0
ZINC000008829785	58	29	23	2	4	0
Imatinib	18	2	0	6	8	2

### 3.4 Molecular dynamics simulation

Root-mean-square deviation and the potential energy of these ligand-PDGFRA complexes were analyzed and used as parameters to evaluate their stability. The results show that the RMSD and potential energy of compound 1, 2-PDGFRA complex reach an equilibrium trajectory at 100 ps and remain stable over time after that. It is proved that these two complexes can exist stably in the natural environment (Figures 6D, E).

## 4 Discussion

High-grade gliomas are common and aggressive pediatric and adult brain tumors. It is estimated that the median overall survival (OS) of adult patients with glioblastoma (GBM), a grade IV glioma, is 12.6 months (Liu et al., 2018) and that for pediatrics with HGG it is 14.1 months (Mackay et al., 2017). It is usually treated with surgery, combined radiotherapy and chemotherapy, and adjuvant temozolomide (TMZ) 6 months after surgery (Stupp et al., 2005). Patients always relapse after adjuvant therapy protocols, which only extend survival by 3 months (Stupp et al., 2009). Around 90% of cases recur, and the prognosis is poorer when HGG recurs (Weller et al., 2013). Most recurrences occur within 2 cm of the margin of the initial tumor, are usually inaccessible by surgery, and respond less well to therapy (Audureau et al., 2018; Aldaz and Arozarena, 2021). Therefore, it is vital to research targeted therapy and develop more targeted drugs to treat HGG.

Targeted therapy is still in the exploratory stage. It is shown that most HGGs demonstrated amplification of PDGFRA-driven signal (Paugh et al., 2011). The ATP-binding site of PDGFRA can be occupied when the PDGFRA inhibitor is in the inactive conformation, preventing substrate phosphorylation and inhibiting downstream signaling (Bauer et al., 2021). Currently, the most studied PDGFRA inhibitors include Dasatinib, Avapritinib, Imatinib, and so on. Moreover, preclinical studies have shown that Imatinib can prevent the PI3K/mTOR signaling pathway or MAPK signaling pathway by docking with PDGFRA. It can effectively inhibit tumor growth, exert anti-tumor activity, and be proven effective in pediatric HGG with PDGF pathway alterations (Schwark et al., 2022). In line with this, a small RCT study found that patients with glioblastoma responded frequently to the combination of hydroxyurea and imatinib (Joensuu et al., 2005; Mantica et al., 2018). This study selected Imatinib as the reference drug. However, Imatinib has significant therapeutic limitations, with developmental toxicity and the risk of intratumoral hemorrhage as side effects (Schwark et al., 2022). The screening of more desirable inhibitors of PDGFRA is therefore essential for the treatment of HGG.

Our study used DS 2019’s six modules (LibDock, ADME, TOPKAT, CDOCKER, 3D-QSAR, and molecular dynamics simulation) to screen and identify ideal inhibitors of PDGFRA. The molecular conformation, pharmacological and toxicological properties, binding affinity, and stability were analyzed. And several potential superior inhibitors of PDGFRA were found with reasonable pharmacological and toxicological properties compared with Imatinib, which lays a solid foundation for drug development of PDGFRA inhibitors and HGG therapy.

For virtual screening, 17,931 named, natural, and purchasable compounds were downloaded from the ZINC15 database. Energy optimization and conformational stability were evaluated using the LibDock score. LidDock’s score is influenced by energy optimization and conformational stability, so the higher the score, the better. Using DS 2019’s LibDock module, we selected 9,842 compounds that were considered to have a high affinity for PDGFRA. Additionally, 3,229 compounds had higher LibDock scores than the reference inhibitor Imatinib (LibDock score: 103.104). In addition, the top 20 compounds scored by the LibDock module were selected for further research.

To evaluate pharmacological properties and toxicology of molecules, ADME and TOPKAT modules were applied. The results indicate that compounds 1 (ZINC000008829785) and 2 (ZINC000013377891) are ideal inhibitors of PDGFRA. Compounds 1 and 2 dissolve well in water, indicating that they can be readily absorbed by the body. Additionally, they show no hepatotoxicity or inhibition of CYP2D6, an enzyme that plays a key role in drug metabolism. Furthermore, three toxicity indices, including Ames mutagenicity, rodent carcinogenicity, and developmental toxicity potential, are within reasonable safety limits. This indicates that they may be used in drug development. However, we cannot assume categorically that the other compounds do not have potential drug development applications as PDGFRA inhibitors. It is possible to design specific groups and atoms in order to alter pharmacological and toxicological properties. In some cases, these compounds may also show their potential value in drug development when designed in a certain way. The compounds 1 and 2 were found to be potential inhibitors of PDGFRA. We also analyzed the precise interaction and combination between compounds 1, 2, and PDGFRA.

Additionally, the CDOCKER module was applied to evaluate the chemical bonding and interaction mechanisms of the ligand- PDGFRA complex. In this procedure, CDOCKER interaction energy of complex of PDGFRA with compounds 1, 2, and Imatinib was calculated separately. The higher absolute value of CDOCKER interaction energy means higher stability and affinity of ligand -PDGFRA complex. Compound 1, 2 -PDGFRA complex was proved to be more stable and tighter for their higher absolute value of CDOCKER interaction energy than the reference ligand Imatinib (−34.6412 kcal/mol). Moreover, the interactions and combinations between compounds 1, 2, Imatinib, and PDGFRA were also shown in two-dimensional and three-dimensional structures (Figures 2, 3). In this step, Schrodinger was also used to illustrate the interaction between the ligand and amino acid residues in the protein binding pocket (Figure 3). It is interesting to note that these selected molecules as well as Imatinib overlap a lot at the location of the PDGFRA binding pocket and form bonds with identical amino acid residues (Figure 5). For example, compound 1 and Imatinib form bonds with the identical amino residues VAL-607, ALA-625, and compound 2 and Imatinib form bonds with VAL-607, ALA-625, CYS-677, LEU-825, and LEU-599. The essentially identical binding and interaction patterns suggest that they may have the same inhibitory effect on PDGFRA. Furthermore, VAL-607 and ALA-625 bond in the protein-binding pocket in binding all three small molecules to PDGFRA, which may play a vital role in the structural, and functional domain. Moreover, the binding of amino acids in the binding pocket may be our new criterion for assessing binding capacity. In addition, pharmacophore is the physical and chemical characteristics and spatial arrangement of ligands required for molecular recognition by biomacromolecules. These pharmacodynamic signatures are the active sites of ligand and receptor interactions. Compounds 1 and 2 showed several hydrogen acceptors, hydrophobic centers, and hydrogen donors with the 3D-QSAR module, which indicated that these two molecules are pharmacologically active and have the potential to be developed as inhibitors of PDGFRA. In future research, diverse specific groups can be added to the two compounds to optimize the drug, thus enhancing its efficacy, and making it a perfect PDGFRA inhibitor.

Finally, the molecular dynamics simulation module appraised the stability of ligand-PDGFRA in the natural environment. As parameters for evaluating the stability of these ligand-PDGFRA complexes, RMSD and potential energy were analyzed. The results show that the RMSD and potential energy of compound 1, 2-PDGFRA complex reach an equilibrium trajectory at 100 ps and remain stable over time after that. These two complexes can exist in the natural environment stably.

Even though this study was carefully designed and accurately measured, some limitations remain. There is no literature report on ZINC000008829785 and ZINC000013377891 in the treatment of glioma. As a result, subsequent studies can directly focus on refining and improving the lead compounds chosen in this study. Further prospective studies are needed to validate our findings since the nomogram is based on retrospective studies.

## 5 Conclusion

This study is significant for screening ideal lead compounds and is a critical step in PDGFRA inhibitor drug design. It provides a solid foundation for future drug designation and development. Our calculations suggest these two molecules (ZINC000008829785 and ZINC000013377891) might serve as ideal inhibitors of cancer through a series of advanced technical calculations. Additionally, this study provides practical guidance and technical means for screening potential therapeutic compounds. Drug development could be aided by this advanced approach in the future. This study provides screening of targeted drugs for HGG patients and improves their prognosis.

## Data availability statement

The original contributions presented in this study are included in the article/supplementary material, further inquiries can be directed to the corresponding author.

## Author contributions

SW and WY drafted the manuscript and contributed to editing and revision. LY, MQZ, LP, WM, and MX downloaded datasets and conducted a bioinformatic analysis. FP, MHZ, and HZ performed the analysis of the results. BY, YL, XZ, HS, JC, and JL contributed to figures and tables. YG substantively edited the manuscript. All authors read and agreed to the final version of this manuscript.
